# Salt Stress Accelerates Tyrosine Depletion by *Enterococcus lactis* KUST2812: Multi-Omics Insights into Metabolic Remodeling

**DOI:** 10.3390/foods15183190

**Published:** 2026-09-09

**Authors:** Xiaoqi Gong, Qingyu Ma, Yujie Zhong, Zhijia Liu, Chuanqi Chu, Junjie Yi, Tao Wang

**Affiliations:** 1 Faculty of Food Science and Engineering, Kunming University of Science and Technology, Kunming 650500, China; gxqkmlg@163.com (X.G.); zyj0048@163.com (Y.Z.); zhijia_liu@outlook.com (Z.L.); chuanqichu@aliyun.com (C.C.); junjieyi@kust.edu.cn (J.Y.); 2 Yunnan Key Laboratory of Plateau Food Intelligent Processing, Kunming 650500, China; 3 International Green Food Processing Research and Development Center of Kunming City, Kunming 650500, China; 4 School of Biological and Food Engineering, Honghe University, Mengzi 661199, China; maqingyu0323@163.com

**Keywords:** *Enterococcus lactis*, broad bean paste, white spots, tyrosine catabolism, salt stress, multi-omics

## Abstract

*Enterococcus lactis* KUST2812 is a halotolerant strain isolated from broad bean paste (BBP). It shows strong potential as a safe fermentation starter due to its high survival rate (98.32% in 18% NaCl), absence of hemolytic activity, and susceptibility to clinically relevant antibiotics, with resistance limited to intrinsic traits. Growth and metabolic dynamics suggest that salt stress decouples growth from tyrosine catabolism: while 6% NaCl significantly inhibited bacterial cell growth, tyrosine depletion occurred within 12 h under 6% NaCl, compared with the 96 h required under non-saline conditions. Multi-omics analyses suggest a molecular network associated with this tyrosine conversion under salt stress. Two tyrosine decarboxylase genes (*tdc*) were upregulated by 1.85- and 6.50-fold, respectively. The tyrosine-specific transporter gene *tyrP* was upregulated 4.99-fold, and the protein synthesis-associated gene *tyrS* was upregulated 4.93-fold. These changes collectively support a diversified tyrosine utilization strategy of *E. lactis* KUST2812. Moreover, coordinated responses involving the Na^+^/H^+^ antiporter system, compatible solute transporters, and oxidative stress markers contributed to the metabolic basis for salt adaptation of this strain. Evaluation in BBP fermentation suggested that *E. lactis* KUST2812 may reduce tyrosine accumulation without causing a significant increase in tyramine. These findings offer a potential microbial resource and a theoretical basis for managing tyrosine-related quality issues in fermented foods.

## 1. Introduction

Broad bean paste (BBP) is a traditional Chinese fermented condiment highly regarded for its unique flavor and rich nutritional profile [1]. Driven by complex microbial consortia, BBP fermentation involves dynamic metabolic interactions, with carbohydrate and amino acid metabolism being particularly active during the early and middle stages [2,3,4]. These dynamic microbial processes continuously degrade broad bean substrates into amino acids, organic acids, and flavor compounds [5,6]. Although a high amino acid content is a key quality attribute of BBP, excessive proteolysis, which is often driven by microorganisms like *Aspergillus* spp. [7], can lead to excessive accumulation of tyrosine. Due to its relatively low solubility, excess tyrosine frequently forms white crystals (white spots) on the surface of the product [8,9]. This phenomenon is not unique to BBP but is also prevalent in other fermented products, such as cheese [10], fermented ham [11], and sufu [9].

Tyrosine white spots are often mistaken for mold, severely reducing product appeal. Moreover, tyrosine precipitation imparts an undesirable gritty texture and bitterness, compromising overall sensory quality [12]. Due to the difficulty of controlling tyrosine crystallization, this issue poses substantial challenges for fermented food manufacturers. To mitigate these adverse effects, current research has primarily focused on passively reducing tyrosine production by optimizing fermentation parameters, such as temperature and salinity [9]. However, effective biological strategies for the active catabolism of accumulated tyrosine in high-salt fermentation systems, particularly microbial intervention, remain largely underexplored. In particular, how salt stress modulates the tyrosine metabolic pathway at the molecular level in halotolerant food-derived bacteria has not been characterized.

Given the existing research gap, our previous efforts focused on isolating bacterial strains from BBP that possess both high salt tolerance and the ability to catabolize tyrosine. Among the isolates, *Enterococcus lactis* KUST2812 exhibited excellent application potential. Unexpectedly, salt stress enhanced tyrosine catabolism while simultaneously inhibiting cell growth. This enhancement in catabolic efficiency was substantial compared to normal conditions, even though growth was significantly suppressed. This phenomenon, characterized by suppressed growth yet enhanced metabolic activity, represents an atypical physiological state whose molecular mechanism remains unclear.

Therefore, this study aims to systematically evaluate the safety and starter culture potential of *E. lactis* KUST2812 and elucidate the molecular mechanisms underlying its efficient tyrosine catabolism under salt stress. Specifically, we comprehensively assessed the salt tolerance and safety of this strain and employed a multi-omics approach combining whole-genome sequencing, transcriptomics, and metabolomics to investigate the complex metabolic pathways and regulatory networks involved in this salt-associated metabolic response. Overall, our findings provide a novel microbial resource and a theoretical foundation for managing tyrosine accumulation in high-salt fermented foods.

## 2. Materials and Methods

### 2.1. Screening of Halotolerant Bacterial Strains

Halotolerant strains were isolated from BBP samples (Chuxiong Yunquan Sauce Co., Ltd., Chuxiong, China). Samples were homogenized, inoculated into enrichment medium containing 10% NaCl, and incubated at 37 °C with shaking for 72 h. Colonies with distinct morphologies were purified by repeated streaking. Four isolates were obtained and identified as *E. lactis* KUST2812, *Lactiplantibacillus plantarum* YQ1, *Weissella confusa* KUST3424, and *Bacillus subtilis* KUST4527 by 16S rRNA gene sequencing [13]. These four strains were then subjected to a tyrosine catabolism assay (Section 2.2.2) to evaluate their tyrosine-metabolizing capacities. The enrichment medium composition is provided in Table S1.

### 2.2. Evaluation of Tyrosine Catabolism Capacity

#### 2.2.1. Preparation of Tyrosine Degradation Medium

Tyrosine degradation medium was prepared by adding tyrosine powder (400 mg/L) (Macklin Biochemical, Shanghai, China) to MRS broth after autoclaving (115 °C, 20 min). The tyrosine powder was sterilized under UV light for 3 h before addition. Because MRS broth contains endogenous tyrosine from its complex components (e.g., peptone and beef extract), the actual initial tyrosine concentration was determined by sampling and analysis for each experiment to account for batch-to-batch variations.

#### 2.2.2. Tyrosine Catabolism Assay

The four halotolerant strains obtained in Section 2.1 were individually inoculated into the tyrosine degradation medium to evaluate their capacity for catabolism. Uninoculated medium served as the blank control (UF). Seed cultures were prepared by activating glycerol stocks in growth medium at 37 °C for 24 h, followed by two successive subcultures (12 h each). Cells were washed twice, resuspended to an OD_600_ of 0.6 ± 0.05, and inoculated at 2% (*v*/*v*). After 72 h of fermentation at 37 °C with shaking, samples were collected and stored at −80 °C for tyrosine quantification by high-performance liquid chromatography (HPLC; Vanquish, Thermo Fisher Scientific, Waltham, MA, USA) [14]. HPLC analysis was performed on a Vanquish system (Thermo Fisher Scientific, USA) equipped with an Agilent C18 column (250 mm × 4.6 mm, 5 μm). The mobile phase consisted of 0.1 mol/L sodium acetate (pH 4.0 ± 0.05) in methanol (90:10, *v*/*v*) at a flow rate of 1.0 mL/min. The detection wavelength was set at 280 nm, the column temperature was 30 °C, and the injection volume was 10 μL.

### 2.3. Whole-Genome Sequencing

The whole-genome sequencing of E. lactis KUST2812 was conducted as described previously [15]. Samples were transported under cold chain conditions to Shanghai Majorbio Bio-Pharm Technology Co., Ltd. (Shanghai, China) for library preparation and sequencing. Genomic DNA was sequenced on the Illumina NovaSeq platform using paired-end 150 bp reads (PE150) with an insert size of approximately 400 bp, generating no less than 100× coverage depth. De novo assembly was performed using SOAPdenovo2 v2.04, with multiple K-mer parameters tested to obtain optimal contig assembly. Scaffolds were constructed based on paired-end and overlap relationships, and gap filling was carried out using GapCloser (v1.12-r6). Functional annotation was performed using multiple databases: the NR database (https://www.ncbi.nlm.nih.gov/refseq/about/nonredundantproteins/, accessed on 2 March 2026) for protein sequences, the KEGG database (http://www.genome.jp/kegg, accessed on 2 March 2026) for metabolic pathway analysis, the CARD database (version 3.2.5) for antibiotic resistance gene screening using the Resistance Gene Identifier (RGI) with “Perfect” and “Strict” hit criteria, and the Virulence Factors of Pathogenic Bacteria database (VFDB, http://www.mgc.ac.cn/VFs/, accessed on 2 March 2026) for virulence factor screening with a threshold of ≥90% protein sequence identity and ≥90% query coverage. A phylogenetic tree was constructed using MEGA (version 6.0) via the neighbor-joining method based on 31 housekeeping genes.

### 2.4. Strain Characterization

#### 2.4.1. Salt Tolerance

Activated *E. lactis* KUST2812 was inoculated into MRS broth containing 12%, 14%, 16%, and 18% (*w*/*v*) NaCl and incubated at 37 °C with shaking at 200 rpm for 72 h. Viable cell counts were determined at 0 h and 72 h by the pour-plate method using MRS agar (Tianjin Damao Chemical Reagent Factory, Tianjin, China), with the solidified plates incubated inverted at 37 °C for 48 h, to calculate survival rates.

#### 2.4.2. Hemolytic Activity

*E. lactis* KUST2812 was streaked onto blood agar plates containing defibrinated sheep blood (Hopebio, Guangzhou, China) and incubated at 37 °C for 48 h. Hemolysis was assessed by observing clearance zones around colonies: α-hemolysis (greenish zone), β-hemolysis (clear zone), or γ-hemolysis (no change) [16]. *Staphylococcus aureus* CICC10306 (obtained from the China Center of Industrial Culture Collection, Beijing, China) was used as a positive control.

#### 2.4.3. Antibiotic Susceptibility

The disk diffusion method was employed to assess antibiotic susceptibility, based on previously described procedures [17]. Bacterial lawns of *E. lactis* KUST2812 were prepared on MRS agar plates. Antibiotic disks (BKMAM, Changsha, China) containing penicillin (10 µg/piece), ampicillin (10 µg/piece), streptomycin (10 µg/piece), tetracycline (30 µg/piece), doxycycline (30 µg/piece), erythromycin (15 µg/piece), vancomycin (30 µg/piece), polymyxin B (300 µg/piece), cefazolin (30 µg/piece), and ceftazidime (30 µg/piece) were placed on the surface. After incubation at 37 °C for 48 h, inhibition zone diameters were measured.

### 2.5. Growth and Tyrosine Catabolism Dynamics Under Salt Stress

#### 2.5.1. Growth Under Different NaCl Concentrations

Seed cultures of *E. lactis* KUST2812 were prepared as in Section 2.2.2 and inoculated (2%, *v*/*v*) into MRS broth containing 0–10% NaCl. Aliquots (0.2 mL) of the seed culture were transferred to 96-well plates, and growth curves were monitored using an automated microbial growth analyzer (BioTek LogPhase 600, Agilent Technologies, Santa Clara, CA, USA).

#### 2.5.2. Tyrosine Catabolism Under Salt Stress

Tyrosine degradation medium containing 0–10% NaCl was inoculated with 2% seed culture and incubated with shaking (37 °C, 200 rpm). Samples were collected at 0, 6, and 12 h, and on days 1, 2, 4, 7, and 12. Tyrosine concentrations were measured as described in Section 2.2.2.

### 2.6. Scanning Electron Microscopy (SEM)

Based on the trends of tyrosine catabolism under different NaCl concentrations described in Section 2.5.2, 6% NaCl was selected as the salt stress condition (S), while 0% NaCl served as the control (C). The 6% NaCl concentration falls within the range where tyrosine was no longer detected, achieved within 12 h, and was therefore chosen for mechanistic investigation. This concentration provides sufficient salt stress while maintaining efficient catabolic activity. Although the salinity of broad bean paste fermentation typically ranges from 12% to 18%, the mechanistic insights obtained at 6% NaCl remain relevant for understanding the regulatory principles of salt-enhanced catabolism, with subsequent validation under industrial conditions presented in Section 2.11. Cells cultivated in tyrosine degradation medium with 0% or 6% NaCl for 12 h were harvested, washed three times with sterile water, and fixed in 2.5% glutaraldehyde for 12 h. After fixation, cells were dehydrated in a graded ethanol series (30% to 100%) and dried. Samples were sent to Zhong Cheng Characterization Laboratory (Chengdu, China) for SEM observation [18].

### 2.7. Metabolome Detection

Based on the time-course analysis of tyrosine catabolism (Section 2.5.2), the 12 h time point was selected, as it represented the maximal divergence in catabolism rates between the control (0% NaCl) and salt-stressed (6% NaCl) conditions. Seed cultures were inoculated (2%, *v*/*v*) into tyrosine degradation medium containing 0% or 6% NaCl and fermented for 12 h. To obtain extracellular metabolites, the supernatant was collected after centrifugation (1000× *g*, 10 min), immediately flash-frozen in liquid nitrogen, and stored at −80 °C. The cell pellets, washed three times, were used for intracellular metabolite extraction. The intracellular (I) and extracellular (E) samples from the control group and the 6% NaCl-stressed group (I6 and E6) were transported under cold chain conditions to Majorbio Bio-Pharm Technology Co., Ltd. for untargeted metabolomics analysis. Following mass spectrometry, raw data were processed using Progenesis QI software (version 3.0, Waters, Milford, MA, USA). Metabolites were identified by searching against the HMDB (http://www.hmdb.ca/, accessed on 25 March 2026) and LipidBlast (https://fiehnlab.ucdavis.edu/projects/LipidBlast, accessed on 27 March 2026) databases. Principal component analysis (PCA) and orthogonal partial least squares discriminant analysis (OPLS-DA) were conducted using MetaboAnalyst 4.0. Model validation for OPLS-DA was performed using 7-fold cross-validation, with R^2^Y and Q^2^ values reported, and 200 permutation tests were conducted to assess model robustness. Variable importance in projection (VIP) scores were calculated. Differentially expressed metabolites (DEMs) were identified based on VIP > 1 and *p* < 0.05. Metabolites with |log_2_FC| > 1.5 were prioritized for detailed interpretation to focus on the most pronounced abundance changes while reducing the number of variables for downstream analysis. FDR correction was not applied to the metabolomic data; consequently, the metabolomic findings are interpreted as exploratory and hypothesis-generating rather than confirmatory. The key metabolite changes identified in this study are supported by independent transcriptomic and RT-qPCR validation. The DEM data were then uploaded to the OmicStudio platform (https://www.omicstudio.cn/, accessed on 27 March 2026) for heatmap visualization. Concurrently, the data were imported into MetaboAnalyst (version 4.0) for functional enrichment analysis using the KEGG database, mapping the DEMs to the metabolic network of Enterococcus lactis. Bubble plots were generated to visualize enriched functions.

### 2.8. Transcriptomic Analysis

Cell pellets collected from the 12 h fermentation as described in Section 2.7 were used for transcriptomic analysis. Transcriptomic analysis was performed following a previous protocol [19]. Cell pellets were washed three times, flash-frozen in liquid nitrogen, and stored at −80 °C. Samples from control (C) and salt-stressed (S) groups were sent to Majorbio Bio-Pharm Technology Co., Ltd. (Shanghai, China) for RNA extraction and sequencing. Raw reads were aligned to the publicly available *E. lactis* reference genome (GCF_019343125.1) using Bowtie2 (version 2.5.4), and gene expression levels were quantified using RSEM (version 1.3.3). Key differentially expressed genes identified in this analysis were independently validated by RT-qPCR (Section 2.9). Differentially expressed genes (DEGs) were identified using DESeq2 (version 1.42.0) with *padj* < 0.05 and |log_2_FC| ≥ 1 as the threshold. Functional enrichment was analyzed using the Kyoto Encyclopedia of Genes and Genomes (KEGG, version 2023.09), with significance set at *p* < 0.05 after Bonferroni correction. Visualization (scatter plots, heatmaps) and annotation were performed on the Majorbio Cloud platform (https://cloud.majorbio.com/, accessed on 27 March 2026).

### 2.9. RT-qPCR Analysis

To validate the transcriptomic data, six DEGs involved in tyrosine catabolism and salt tolerance were selected for RT-qPCR analysis. The *map* gene was used as the internal reference control based on its stable expression across all experimental conditions in our preliminary validation tests (Ct variation < 0.5 cycles). Gene-specific primers were designed using NCBI Primer-BLAST, and the sequences are listed in Table S2. Reverse transcription was performed using the PrimeScript™ FAST RT reagent Kit with gDNA Eraser (Takara Bio Inc., Kusatsu, Japan, RR092S) according to the manufacturer’s instructions. Briefly, 1 μg of total RNA was treated with 8× gDNA Eraser Premix at 42 °C for 2 min to remove genomic DNA, followed by reverse transcription with 5× RT Premix (containing both Oligo dT Primer and Random 6 mers) at 37 °C for 15 min and 85 °C for 5. The resulting cDNA was diluted 5-fold with RNase-free water before qPCR. RT-qPCR was performed using SYBR^®^ 2× RealStar Fast SYBR qPCR Mix (Takara Bio Inc.) on a Bio-Rad CFX96 real-time PCR detection system (Bio-Rad Laboratories, Hercules, CA, USA) in a 20 μL reaction volume containing 2 μL of diluted cDNA template and 0.4 μM each forward and reverse primer. The cycling protocol was 95 °C for 30 s, followed by 40 cycles of 95 °C for 5 s and 60 °C for 30 s, with a melting curve analysis to confirm amplicon specificity. Each reaction was performed in triplicate technical replicates. Relative gene expression levels were calculated using the 2^−ΔΔCt^ method [20].

### 2.10. Pathway-Based Multi-Omics Interpretation

Genomic, transcriptomic, and metabolomic datasets were analyzed in a pathway-based framework using the Majorbio Cloud Platform. The KEGG database was employed as the primary reference to map DEGs and DEMs onto the same pathway framework. Gene–metabolite associations were established by cross-referencing KEGG Orthology (KO) numbers and compound IDs within the same pathways, thus facilitating qualitative linking of transcriptional and metabolic changes. The analysis was primarily based on pathway-level mapping using KEGG, rather than quantitative correlation analysis between transcript and metabolite abundances.

### 2.11. Fermented BBP Samples

Given the tyrosine-degrading capability of *E. lactis* KUST2812 identified in previous experiments, this strain was inoculated into a BBP fermentation system to evaluate its actual impact on product quality. BBP samples collected after four months of fermentation were used as the basal substrate. This time point was selected because it corresponds to a period of active microbial metabolism, making it an ideal substrate for evaluating starter cultures. Moreover, using this partially fermented broth as the base material allowed for a shortened fermentation period and facilitated a more rapid assessment of the microbial effects. Samples collected at 0 h were designated as the blank control (C) to represent the initial state of the fermentation system. The seed culture (OD_600_ = 1.0) was inoculated at 3% (*v*/*v*) into the BBP system as the experimental group (El), and a naturally fermented group without inoculation (NF) was incubated under the same conditions as a parallel control. After 15 days of fermentation at 37 °C under static conditions, the levels of tyrosine and tyramine in the fermented BBP juice (FBPJ) were measured. Tyrosine content was determined following the method described in Section 2.2.2, while tyramine content was analyzed according to a previously described protocol [21].

### 2.12. Statistical Analysis

All experiments were performed in biological triplicate, and the data are expressed as the mean ± standard deviation. Statistical analyses were conducted using Origin software (2024 edition). Before parametric tests, data normality was assessed using the Shapiro–Wilk test, and homogeneity of variances was evaluated using Levene’s test. All datasets met the assumptions for parametric analysis. Comparisons between different groups were made using a *t*-test (two groups) or one-way analysis of variance (ANOVA), followed by the Holm–Šidák post-hoc test. Differences were considered statistically significant at *p* < 0.05. For HPLC and RT-qPCR analyses, three technical replicates were performed for each biological sample to monitor instrument stability; the average of the technical replicates was used for statistical comparison. For metabolomic and transcriptomic analyses, single measurements were performed per biological sample, with data quality ensured by QC sample monitoring (metabolomics) and high Q30 scores (transcriptomics). All statistical analyses were based on biological replicates (*n* = 3). For the degradation kinetics, one-way ANOVA was applied at each time point; a two-way ANOVA would more comprehensively evaluate concentration–time interactions, and this limitation should be considered when interpreting the results. Schematic diagrams were generated using BioRender (https://www.biorender.com/).

## 3. Results and Discussion

### 3.1. Isolation of Salt-Tolerant Strains Capable of Metabolizing Tyrosine

**Figure 1:**
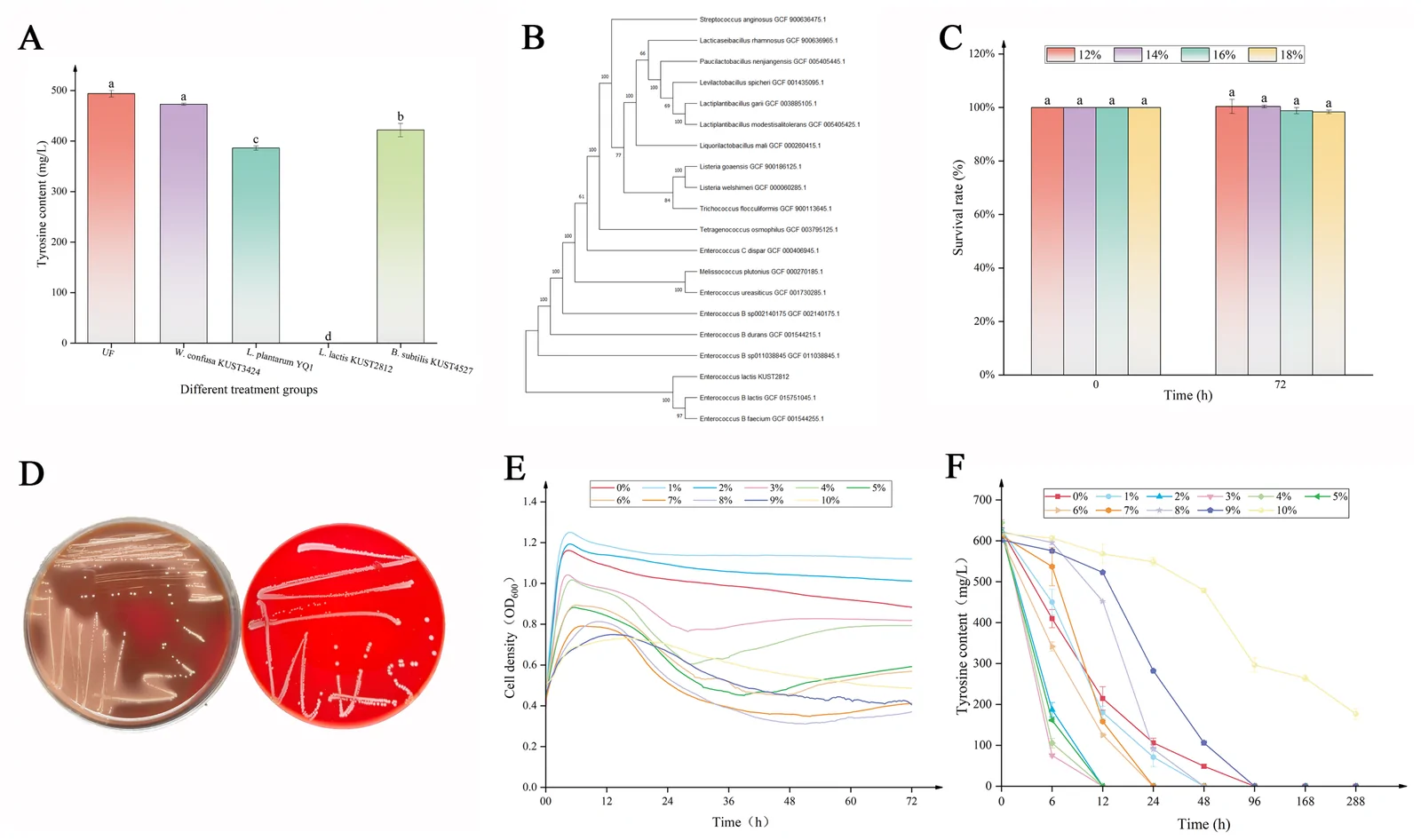
Characteristics, salt tolerance, and physiological responses of *E. lactis* KUST2812 under salt stress. (**A**) Tyrosine catabolism capacity of different strains after 72 h of fermentation (UF: uninoculated blank control). Different lowercase letters indicate statistically significant differences among strains (*p* < 0.05). (**B**) Phylogenetic tree of *E. lactis* KUST2812. (**C**) Salt tolerance of *E. lactis* KUST2812. Different lowercase letters indicate statistically significant differences among strains (*p* < 0.05). (**D**) Hemolytic activity of *S. aureus* CICC10306 (positive control, **left**) and *E. lactis* KUST2812 (**right**). (**E**) Growth curves under different NaCl concentrations. (**F**) Tyrosine catabolism kinetics under different NaCl concentrations.

Four strains capable of surviving in a high-salt environment (10% NaCl) were isolated from traditional BBP. Based on 16S rRNA gene sequencing, these strains were identified as *E. lactis* KUST2812, *Lactiplantibacillus plantarum* YQ1, *Weissella confusa* KUST3424, and *Bacillus subtilis* KUST4527. Their tyrosine-metabolizing capabilities were subsequently evaluated in a medium supplemented with exogenous tyrosine. After 72 h of fermentation, *E. lactis* KUST2812 consumed the available tyrosine to below the detection limit, whereas significant residual tyrosine was detected in the cultures of the other three strains (Figure 1A). This result demonstrated that *E. lactis* KUST2812 possesses a superior tyrosine catabolism capacity among the candidate strains, making it the most suitable candidate for further investigation. This finding aligns with previous studies highlighting the robust tyrosine-degrading ability of *Enterococcus* species, further validating their role as key microorganisms in tyrosine metabolism research [22,23,24].

### 3.2. Whole-Genome Sequencing Analysis

To elucidate the genetic basis and metabolic potential of *E. lactis* KUST2812, whole-genome sequencing and comprehensive bioinformatic analyses were performed. A phylogenetic tree based on 31 housekeeping genes supported the identification of the strain as *E. lactis* (Figure 1B). The assembled genome comprised a single circular chromosome of 2,921,801 bp, with a total coding sequence length of 2,503,674 bp and an overall GC content of 37.89% (Figure S1). Gene prediction identified 2915 coding sequences with an average gene length of 858.89 bp. Furthermore, the genome also contained 63 tRNA genes, 7 rRNA genes (one 16S rRNA, one 23S rRNA, and five 5S rRNA), and 44 sRNA genes.

To understand the functional repertoire of the strain, genes were annotated using multiple databases. Annotation against the KEGG database assigned 1983 genes to six major functional categories: cellular processes, environmental information processing, genetic information processing, human diseases, metabolism, and biological systems (Figure S2). Notably, “metabolism” was the most abundant category, predominantly featuring subcategories such as global and overview maps, carbohydrate metabolism, and amino acid metabolism. Concurrently, annotation against the NR database successfully mapped 2911 genes. From this dataset, 51 genes specifically related to salt tolerance were identified. These genes were classified into five functional categories: ion transport, membrane structure and modification, osmolyte synthesis and transport, signal transduction, and stress response and protein homeostasis (Table 1). Additionally, screening against the CARD database revealed 170 potential antibiotic resistance genes, providing a genetic baseline for subsequent safety evaluations.

### 3.3. Salt Tolerance and Safety Characteristics

**Figure 2:**
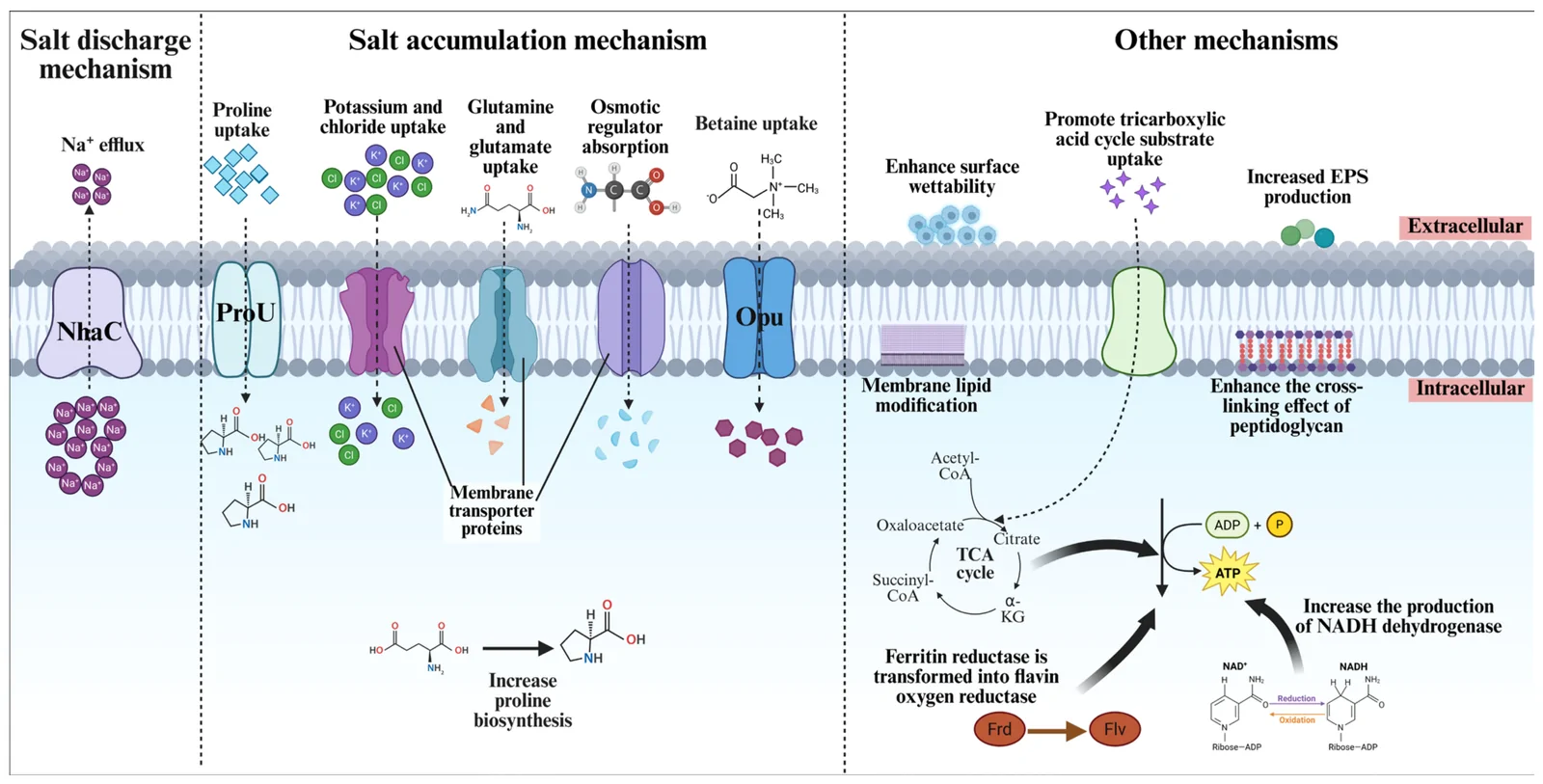
Schematic diagram of salt tolerance mechanisms in *Enterococcus lactis* KUST2812. Schematic diagram of salt tolerance mechanisms in *E. lactis* KUST2812. Arrows indicate regulatory or metabolic fluxes; different colors distinguish functional modules (ion transport, osmolyte synthesis, membrane modification, signal transduction, and stress response) as labeled.

The survival rate of *E. lactis* KUST2812 under different NaCl concentrations is presented in Figure 1C. Remarkably, after 72 h of cultivation in medium containing 18% NaCl, the strain still maintained a survival rate of 98.32%, demonstrating exceptional halotolerance. As illustrated in Figure 2 and Table 1, genomic analysis revealed that the salt adaptation strategies of this strain primarily involve ion efflux systems (e.g., Na^+^/H^+^ antiporters), ion accumulation strategies (e.g., accumulation of compatible solutes such as proline), and other adaptations such as membrane modification [25]. The Na^+^/H^+^ antiporter system on the cell membrane represents a direct mechanism for mitigating salt stress by actively extruding intracellular Na^+^ in exchange for H^+^, thereby maintaining intracellular osmotic pressure and pH homeostasis [13]. Correspondingly, whole-genome analysis identified three genes encoding Na^+^/H^+^ antiporters in *E. lactis* KUST2812, including two *nhaC* genes and one *nhaK* gene (Table 1). Furthermore, the accumulation of compatible solutes (e.g., glycine betaine, proline, glutamate, and K^+^) represents another important strategy for osmotic adjustment in bacteria [26,27]. Genomic mining uncovered multiple transporter genes in *E. lactis* KUST2812 for these solutes, including *proV* and *proW* (ProU system) and *opuA* and *opuC* (Opu family) [27], alongside proline biosynthesis genes (*proA* and *proB*) [28]. Additionally, membrane modification is another key strategy for coping with salt stress. Modulating membrane lipid composition to maintain membrane fluidity, regulating membrane surface properties, and secreting exopolysaccharides (EPS) can effectively preserve membrane structural stability and alleviate salt-induced damage [29].

Beyond environmental adaptability, safety is a prerequisite for starter cultures in the food industry. No hemolytic activity was observed for *E. lactis* KUST2812, in contrast to the β-hemolytic positive control *Staphylococcus aureus* CICC10306 (Figure 1D) [30]. Antibiotic susceptibility testing revealed that the strain was sensitive to penicillin, ampicillin, tetracycline, doxycycline, and vancomycin; intermediately resistant to streptomycin and cefazolin; and resistant to erythromycin, polymyxin B, and ceftazidime (Table S3). Although several vancomycin resistance-associated genes (*vanR*, *vanS*, *vanH*, *vanZ*, *vanA*, and *vanB*) were identified, their low homology and sequence coverage suggest they are incomplete fragments with no functional relevance. In contrast, the vancomycin target enzyme D-Ala-D-Ala ligase (encoded by the *ddl* gene) was identified with high integrity (82.2% identity, 98.3% coverage), consistent with the observed vancomycin sensitivity [31,32,33]. Erythromycin resistance genes (*msrC*, *efmA*, and *efrA*) were also detected, and the presence of these efflux pumps may contribute to the strain’s intermediate resistance to cefazolin and ceftazidime [34]. Overall, these findings indicate that *E. lactis* KUST2812 is non-hemolytic, susceptible to clinically relevant antibiotics.

It should be noted that the genus *Enterococcus* has been the subject of safety debate in food applications, primarily due to the presence of antibiotic resistance and virulence determinants in certain species, particularly *E. faecalis* and *E. faecium* [35]. However, recent genomic surveys have demonstrated that the distribution of these determinants is highly species- and strain-dependent, with many food-derived *Enterococcus* strains carrying no such traits [36]. For *E. lactis* KUST2812, multiple lines of evidence support a favorable preliminary safety profile: the absence of hemolytic activity; the lack of functional vancomycin resistance genes (as confirmed by low homology and coverage of van gene fragments), with the intact target gene *D-Ala-D-Ala* ligase consistent with the observed vancomycin susceptibility; susceptibility to clinically relevant antibiotics (penicillin, ampicillin, tetracycline, doxycycline, and vancomycin); and the absence of high-confidence virulence determinants. Specifically, systematic screening of the genome for known Enterococcus virulence-associated genes—including adhesion factors (*acm*, *scm*, *sgrA*, *ecbA*, *fnm*, *sagA*, *bepA*), biofilm formation genes (*empA*, *empB*, *empC*), pili gene clusters (fms family), and other virulence markers (*hylEfm*, *esp*, *cylA*, *asa1*, *gelE*, *ace*, *efaA*)—identified no hits with both identity and coverage ≥90%, a stringent threshold widely used in whole-genome sequencing-based safety assessment to distinguish functional homologs from non-functional similarities [37]. These combined phenotypic and genotypic assessments provide a favorable preliminary safety profile for *E. lactis* KUST2812 and support its further evaluation as a candidate starter culture. A comprehensive strain-specific safety assessment, including standardized MIC testing, distinction between intrinsic and acquired resistance, and analysis of mobile genetic elements, remains necessary before industrial application.

### 3.4. Growth and Catabolism Dynamics Under Salt Stress

To further characterize the physiological response of *E. lactis* KUST2812 to varying salinities, its growth dynamics were monitored across a range of NaCl concentrations (0–10%) (Figure 1E). The strain typically reached its exponential growth state at 4–5 h. Notably, mild salinity (1% and 2% NaCl) slightly stimulated cell proliferation compared to the non-stressed control (0% NaCl). However, as NaCl concentrations increased from 3% to 10%, bacterial growth was progressively inhibited, consistent with the well-documented suppressive effects of hyperosmotic stress on cell division [38,39].

Concurrently, the tyrosine catabolism profiles exhibited a striking and paradoxical trend (Figure 1F and Table S4). Under the control condition (0% NaCl), tyrosine was no longer detected after 96 h. Remarkably, moderate salt stress significantly accelerated this process: Tyrosine was no longer quantifiable within 48 h at 1% NaCl, and within 12 h at 2–6% NaCl. Under higher salinity (7–9% NaCl), tyrosine was no longer detected at 24 h, 48 h, and 96 h, respectively. At 10% NaCl, tyrosine depletion occurred gradually throughout the entire 288-h (12-day) observation period. These results reveal a seemingly counterintuitive pattern. Although NaCl concentrations above 3% substantially inhibited cell growth, the rate of tyrosine catabolism within the 3–7% NaCl range was higher than that in the control. Similar stress-induced metabolic phenomena have also been documented in other halotolerant bacteria, where environmental stress suppresses growth but enhances specific catabolic or biosynthetic activities. Examples include ectoine production in *Halomonas elongata* DSM 2581T [40] and EPS/IAA production in *Halobacillus trueperi* RSK CAS9 [41]. Notably, those studies focused on industrial or agricultural applications, whereas our work demonstrates this phenomenon in a food-derived strain with direct relevance to fermented food quality control. Collectively, these findings suggest that salt stress redirects metabolic flux, prioritizing specific catabolic pathways over biomass accumulation.

### 3.5. SEM Analysis

**Figure 3:**
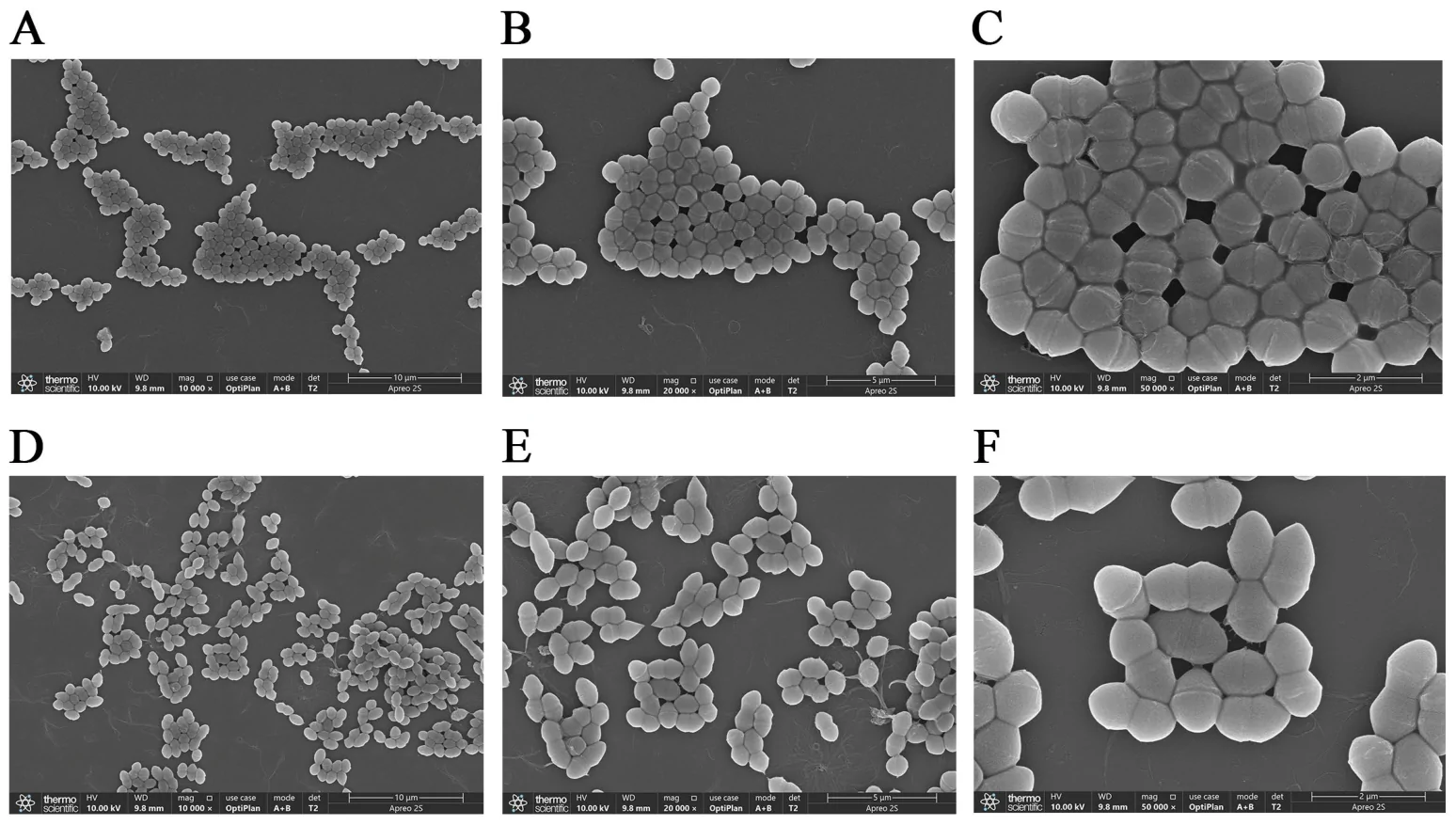
Scanning electron microscopy (SEM) images of *E. lactis* KUST2812 cultured under 0% NaCl (**A**–**C**) and 6% NaCl (**D**–**F**) for 12 h. (**A**–**C**) Scanning electron microscopy (SEM) images of cell morphology under control conditions at ×10,000, ×20,000, and ×50,000 magnification, respectively. (**D**–**F**) SEM images of cell morphology under salt stress at ×10,000, ×20,000, and ×50,000 magnification, respectively.

To elucidate the microscopic impact of the aforementioned salt stress on cellular integrity, SEM was employed to visualize the morphological changes in *E. lactis* KUST2812 cultivated under 0% and 6% NaCl conditions for 12 h. Under normal conditions (Figure 3A–C), cells appeared spherical with smooth surfaces covered by abundant extracellular matrix-like material [18]. Under salt stress (Figure 3D–F), cell morphology was altered: cells shrank and appeared elliptical with pointed ends, while also exhibiting pronounced surface cracking [42]. These changes may result from cell wall detachment and membrane damage, both induced by high osmotic pressure [43].

### 3.6. Metabolomic Analysis

**Figure 4:**
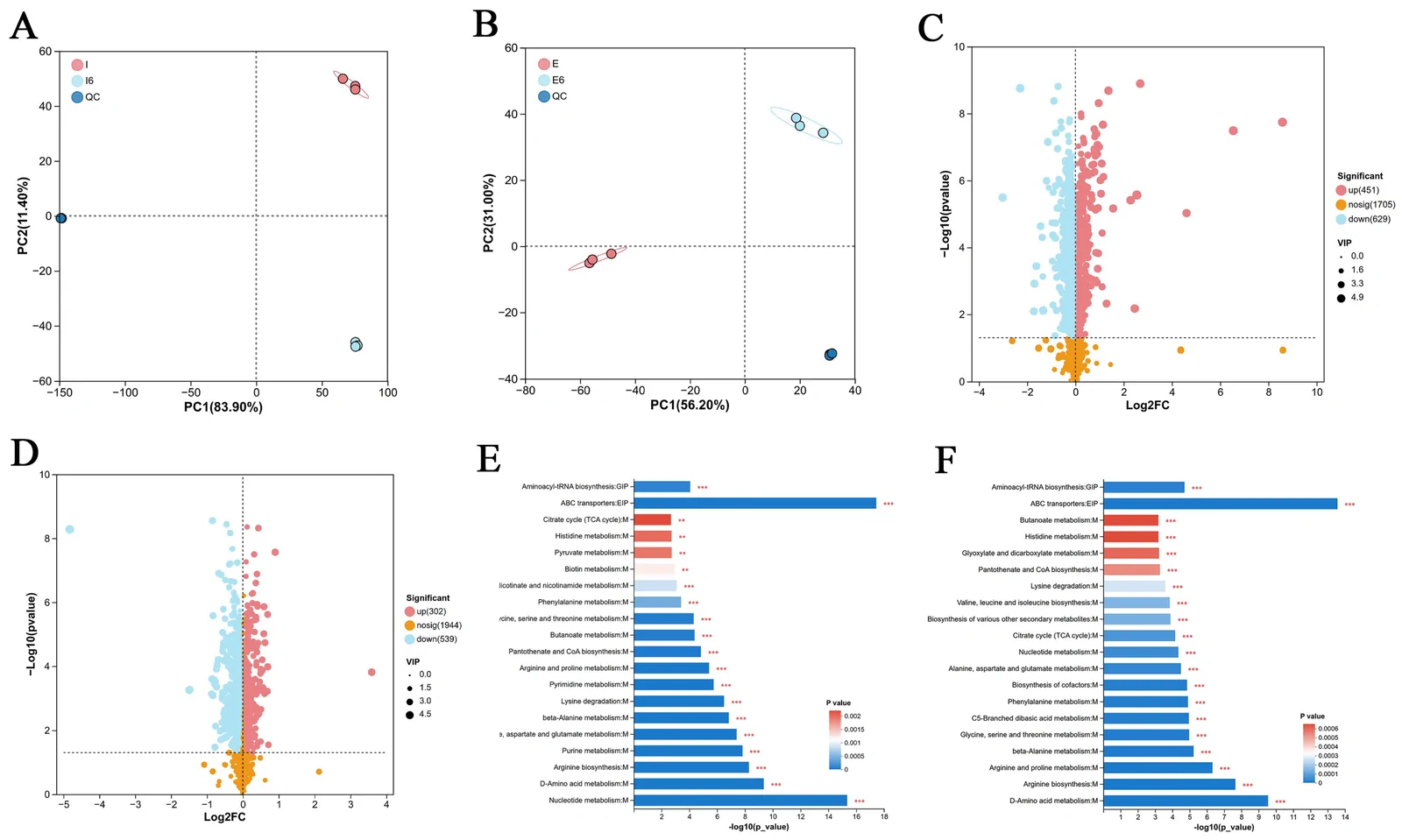
Integrated analysis of differentially expressed metabolites (DEMs) in intracellular and extracellular fractions. (**A**) PCA plot of intracellular metabolites. (**B**) PCA plot of extracellular metabolites. (**C**) Volcano plot of intracellular DEMs. (**D**) Volcano plot of extracellular DEMs. (**E**) KEGG enrichment analysis of intracellular DEMs. (**F**) KEGG enrichment analysis of extracellular DEMs. In (**E**,**F**), the *x*-axis represents the enrichment ratio, defined as the number of DEMs annotated to a given pathway divided by the total number of metabolites in that pathway in the species. The *y*-axis indicates the enriched KEGG pathway names. “**” corresponds to *p* < 0.01, “***” corresponds to *p* < 0.001.

To elucidate the immediate biochemical consequences of salt stress on tyrosine metabolism, an untargeted metabolomic analysis was first performed on both intracellular (I) and extracellular (E) fractions. Metabolomic PCA revealed distinct separation between the control and 6% NaCl-stressed groups for both intracellular (I6 vs. I) and extracellular (E6 vs. E) metabolites (Figure 4A,B). The first principal component (PC1) accounts for the major variance between the two experimental conditions, indicating that salt stress represents the dominant source of metabolic divergence in both intracellular and extracellular fractions. This suggests that salt stress not only reprograms internal metabolic fluxes but also significantly alters the distribution of metabolites between intracellular and extracellular compartments.

Quantitative analysis identified a vast array of DEMs, including 1080 intracellular DEMs (451 upregulated, 629 downregulated) and 841 extracellular DEMs (302 upregulated, 539 downregulated) (Figure 4C,D). Among these, subsequent interpretation focused on metabolites with the most pronounced changes (|log_2_FC| > 1.5), particularly those directly relevant to tyrosine metabolism and salt-adaptive pathways. KEGG enrichment analysis revealed that intracellular DEMs were enriched in 109 pathways (42 significantly enriched, *p* < 0.05), and extracellular DEMs in 103 pathways (48 significantly enriched, *p* < 0.05). The top 20 enriched pathways for both fractions (Figure 4E,F) were primarily associated with carbohydrate metabolism (e.g., glycolysis, TCA cycle), nucleotide metabolism (e.g., purine and pyrimidine metabolism), and diverse amino acid metabolism (e.g., arginine, alanine, aspartate, glutamate, histidine, lysine, proline, and glycine/serine/threonine metabolism). Additionally, pathways related to butanoate metabolism, cofactor biosynthesis, and C5-branched dibasic acid metabolism were significantly enriched. These pathways are functionally interconnected: carbohydrate metabolism provides energy and carbon skeletons for amino acid biosynthesis, while nucleotide metabolism supports nucleic acid repair under oxidative stress. These findings are consistent with previous studies demonstrating that salt stress profoundly modulates carbohydrate and amino acid metabolism [44]. Collectively, these metabolomic profiles confirm that salt stress profoundly modulates the global metabolic network of *E. lactis* KUST2812, orchestrating a complex reallocation of carbon, nitrogen, and energy resources to facilitate osmotic adaptation.

### 3.7. Transcriptomic Analysis and RT-qPCR Validation

**Figure 5:**
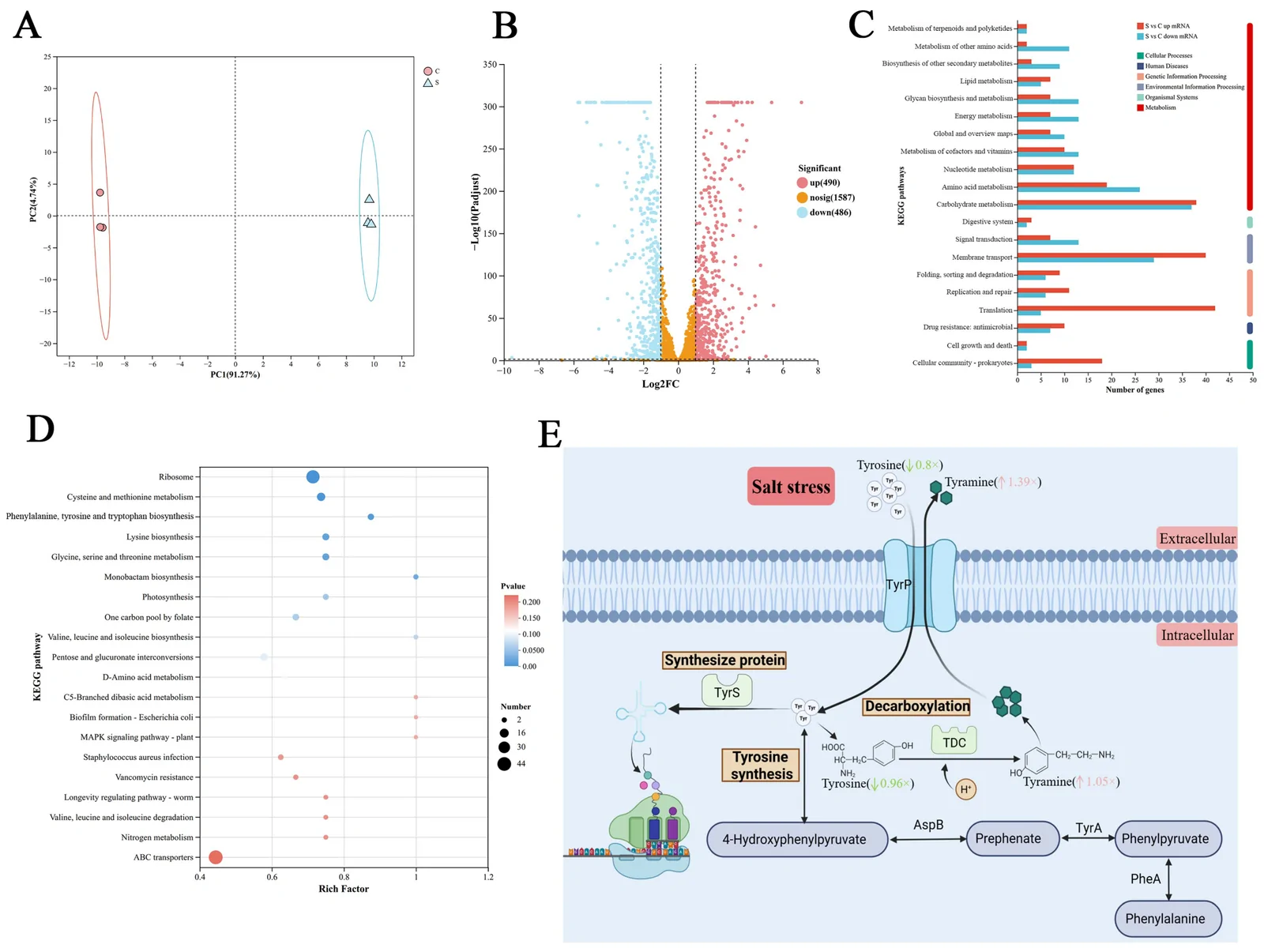
Transcriptomic analysis and a proposed mechanistic model. (**A**) Principal component analysis (PCA) plot of transcriptomic samples. (**B**) Volcano plot of differentially expressed genes (DEGs). (**C**) KEGG functional annotation of DEGs. (**D**) Bubble plot of KEGG pathway enrichment analysis for DEGs. (**E**) Proposed working model of tyrosine metabolism in *E. lactis* KUST2812 under salt stress, constructed from genomic, transcriptomic, and metabolomic data. Extracellular tyrosine is imported via the TyrP transporter and subsequently channels into three metabolic routes: (1) decarboxylation to tyramine, catalyzed by tyrosine decarboxylase (TDC), with tyramine exported to the extracellular compartment; (2) conversion to 4-hydroxyphenylpyruvate via aminotransferase (EC 2.6.1.1), enabling further downstream reactions; and (3) incorporation into protein synthesis through tyrosyl-tRNA synthetase (TyrS). Arrow annotations adjacent to tyrosine and tyramine indicate fold changes in metabolite abundance under salt stress (red: upregulation; green: downregulation).

To delineate the transcriptional basis underlying the salt-induced metabolic remodeling observed above, comparative transcriptomic analysis was conducted between the control (C) and 6% NaCl-stressed (S) groups. The sequencing data exhibited high reliability, with all samples achieving a Q30 quality score above 97.07% (Table S5). PCA revealed a profound transcriptomic divergence between the two conditions, with PC1 and PC2 collectively accounting for 96.01% of the total variance (Figure 5A). PC1, which explained the largest proportion of the variance, clearly separates the control from the salt-stressed samples, confirming that salt treatment is the primary driver of transcriptional reprogramming. This distinct clustering indicates that hyperosmotic stress triggered massive alterations in gene transcription. Specifically, a total of 976 DEGs were identified, including 490 upregulated and 486 downregulated genes (Figure 5B).

Functional classification of the DEGs showed that they were predominantly mapped to carbohydrate metabolism, amino acid metabolism, membrane transport, and translation processes (Figure 5C). This systemic metabolic shift aligns with the physiological adaptations required for salt stress-affected pathways in *Weissella confusa* [44]. Furthermore, KEGG pathway enrichment analysis revealed significant enrichment (*p* < 0.05) of specific pathways crucial for cellular homeostasis and stress response, including ribosome assembly, cysteine and methionine metabolism, phenylalanine/tyrosine/tryptophan biosynthesis, lysine biosynthesis, glycine/serine/threonine metabolism, and monobactam biosynthesis (Figure 5D). The prominent enrichment of these diverse amino acid-related pathways provides a crucial molecular clue for the accelerated tyrosine turnover observed under salt stress.

**Figure 6:**
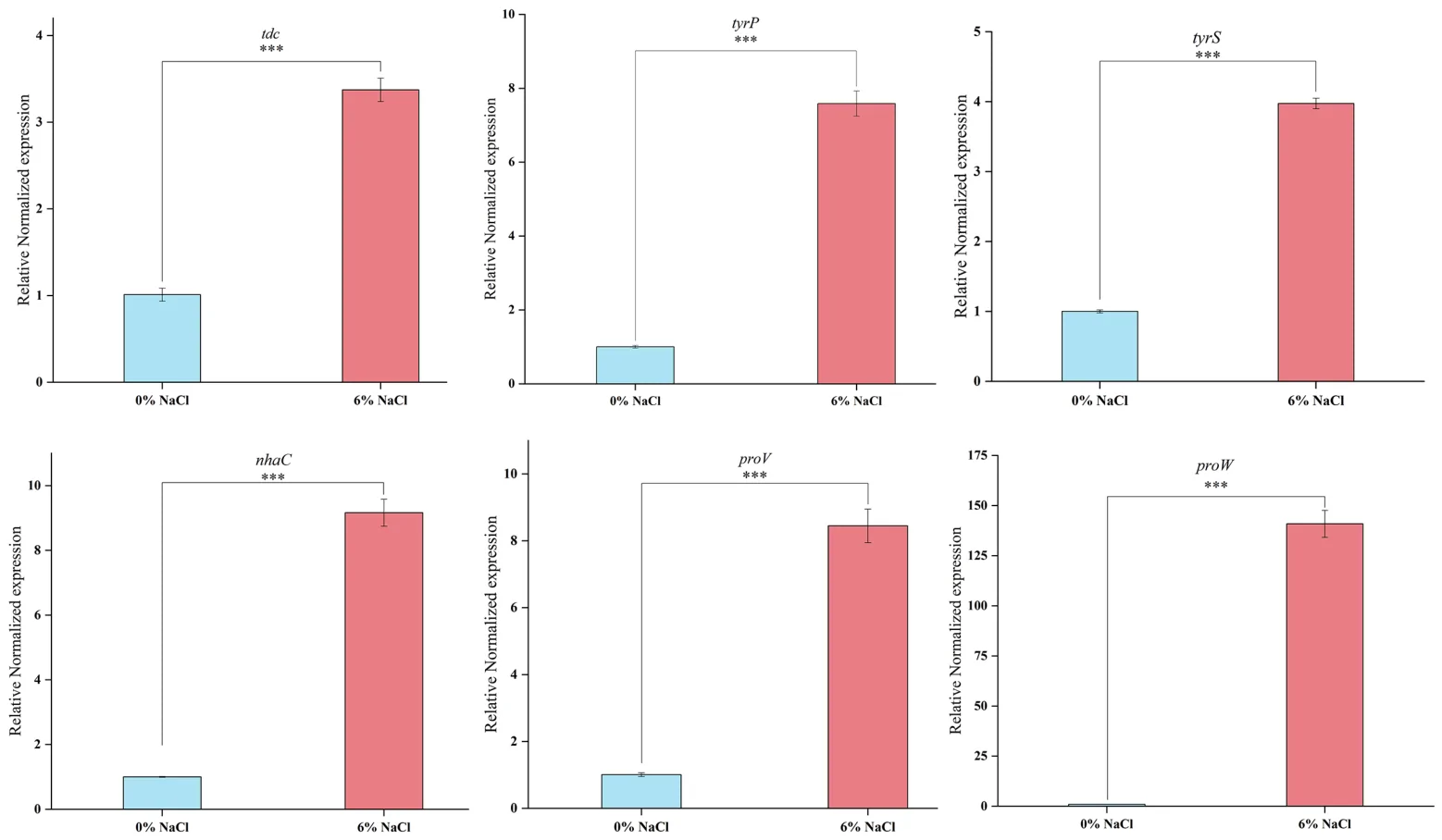
RT-qPCR validation of selected genes involved in tyrosine catabolism and salt tolerance. Relative mRNA expression levels of *tdc*, *tyrP*, *tyrS*, *nhaC*, *proV*, and *proW* were measured. The map gene was used as the internal reference control. The relative fold changes were calculated using the 2^−ΔΔCt^ method. “***” corresponds to *p* < 0.001.

To confirm the reproducibility of the RNA-seq results, six selected DEGs, namely *tdc*, *tyrP*, *tyrS*, *nhaC*, *proV*, and *proW*, were subjected to RT-qPCR analysis. As shown in Figure 6, the expression trends obtained by RT-qPCR were consistent with those from the transcriptomic data, confirming the reliability of the RNA-seq analysis.

### 3.8. Pathway-Based Multi-Omics Interpretation of the Molecular Network Associated with Salt-Enhanced Tyrosine Catabolism

#### 3.8.1. Mechanism of Salt-Enhanced Tyrosine Catabolism

To elucidate the molecular mechanisms associated with the salt-enhanced tyrosine catabolism in *E. lactis* KUST2812, we combined whole-genome, transcriptomic, and metabolomic datasets. Based on KEGG annotation of the genome, three putative pathways for tyrosine metabolism were identified: decarboxylation to tyramine, conversion to 4-hydroxyphenylpyruvate, and incorporation into tyrosyl-tRNA (Tyr) for protein synthesis (Figure 5E). The expression levels of key genes and the abundances of related metabolites were systematically mapped (Table 2 and Table S6). Collectively, these multi-omics data suggest new insights into salt-induced acceleration of a specific tyrosine catabolic pathway in a food-derived halotolerant bacterium, offering a potential theoretical basis for managing tyrosine accumulation in high-salt fermented foods.

The metabolomic data further corroborated the observed salt-enhanced catabolism phenotype (Figure 1F), showing depletion of tyrosine levels under salt stress in both intracellular (FC = 0.96) and extracellular (FC = 0.80) fractions (Table S6). Transcriptomic profiling suggested potential transcriptional drivers for this accelerated depletion: two *tdc* genes, encoding tyrosine decarboxylase (TDC, EC 4.1.1.25) [45], were significantly upregulated under salt stress (1.85-fold and 6.50-fold, respectively). Correspondingly, the decarboxylation product, tyramine, accumulated moderately intracellularly (FC = 1.05) and more markedly extracellularly (FC = 1.39) (Table S6). Concurrently, the gene *tyrP*, encoding a tyrosine-specific transporter capable of bidirectional tyrosine and tyramine transport [46], was markedly upregulated (from 7.49 to 37.38). This coordinated upregulation of the *tdc–tyrP* module is consistent with rapid influx of extracellular tyrosine and the subsequent efficient efflux of intracellularly synthesized tyramine, which may account for the observed extracellular tyramine surge (Table S6). The concurrent upregulation of *tyrS* (encoding tyrosyl-tRNA synthetase, EC 6.1.1.1) is consistent with the hypothesis that accelerated tyrosine decarboxylation may serve a dual purpose: removal of extracellular tyrosine and regeneration of intracellular amino acid pools to support stress-responsive protein synthesis, although transcriptional changes alone do not establish metabolic flux.

While the pathway-based integrated multi-omics analysis suggests the *tdc*–*tyrP* module in salt-enhanced tyrosine conversion, direct biochemical validation is warranted. Future studies should focus on quantifying tyrosine decarboxylase activity in vitro across a gradient of salt concentrations. Additionally, determining whether salt stress induces conformational changes or post-translational modifications in TDC that enhance its catalytic efficiency will be crucial. Such mechanistic insights will provide a vital theoretical foundation for the rational design and application of starter cultures with precisely controlled tyrosine metabolism.

#### 3.8.2. Global Physiological Responses and Metabolic Adaptations to Salt Stress

Beyond the specific activation of tyrosine metabolism, surviving and sustaining this highly active catabolic state under hyperosmotic conditions required broader physiological adaptations. Consequently, *E. lactis* KUST2812 deployed a robust, globally upregulated salt-tolerance network. Two *nhaC* genes, encoding Na^+^/H^+^ antiporters essential for Na^+^ efflux, were significantly upregulated (from 2.68 to 8.75 and 8.13 to 72.91, respectively) (Table 2). In contrast, *nhaK* was not detected in the transcriptome, suggesting that NhaC serves as the primary Na^+^ extrusion system under the tested conditions [47]. Simultaneously, an extensive compatible solute scavenging strategy was activated. The *proV* and *proW* genes, encoding a glycine betaine/proline ABC transporter, were also significantly upregulated (Table 2), consistent with the observed increase in intracellular proline (FC = 1.03) and its decrease extracellularly (FC = 0.96) (Table S6). Moreover, multiple other salt-tolerance-associated genes (*trkA*, *trkH*, *clcA*, *zntA*, *gadC*, *oppD*, *oppA*, *oppB*, *fruA*) were upregulated, and various amino acids (including glutamate, citrulline, ornithine, aspartate, threonine, isoleucine, valine, lysine, and histidine) accumulated intracellularly while decreasing extracellularly (Table S6). These observations support a coordinated strategy of compatible solute uptake to counteract osmotic stress [48].

While the strain effectively managed osmotic balance, the severe metabolic cost of this adaptation manifested as growth inhibition and oxidative stress. UDP-N-acetyl-D-galactosamine (UDP-GalNAc) is an activated nucleotide sugar that serves as a key structural component of bacterial cell wall biosynthesis [49]. Disruption of UDP-GalNAc metabolism has been shown to impair cell wall synthesis and lead to growth retardation [50]. In this study, UDP-GalNAc levels were significantly reduced under salt stress in both intracellular (FC = 0.4) and extracellular (FC = 0.56) fractions (Table S6), consistent with the observed growth inhibition at 6% NaCl (Figure 1E). This suggests that the downregulation of this metabolite may reflect suppressed cell proliferation. This interpretation is consistent with the significant downregulation of *ftsE*, a cell division gene, in the transcriptomic data (Table 2), indicating reduced cell division efficiency under salt stress. In addition, markers of oxidative stress were elevated. 8-Hydroxyguanosine (8-OHG), a marker of RNA oxidation, increased substantially in both intra- and extracellular fractions (FC = 1.17 and 1.41, respectively) (Table S6), which may suggest possible RNA oxidative damage [51]. α-Hydroxybutyric acid (AHB), a metabolic stress marker generated upon depletion of the antioxidant glutathione (GSH) during oxidative stress [52], was also significantly upregulated intracellularly (FC = 1.23), suggesting that the strain experienced an elevated oxidative burden and that its antioxidant defense systems were activated.

Beyond amino acid metabolism, salt stress broadly activated carbohydrate and nucleotide metabolism. Intracellular accumulation of arabinose, D-arabinonic acid, and D-galactosamine suggested activation of pentose and amino sugar metabolism (Table S6). Trehalose, a common compatible solute [53], unexpectedly decreased intracellularly (FC = 0.70) while increasing extracellularly (FC = 1.11) (Table S6). Transcriptomic data showed upregulation of the trehalase gene *treC* (2.35-fold) (Table 2), whereas trehalose biosynthesis genes (*otsA*, *otsB*) and the trehalose exporter (*treT*) were not detected, suggesting that enhanced trehalose catabolism, rather than impaired synthesis, drove its intracellular decline. The concomitant extracellular increase may reflect leakage due to salt-induced cell surface damage, consistent with SEM observations (Figure 3).

Nucleotide metabolites also showed altered abundance under salt stress, with intracellular accumulation of inosine (FC = 1.91), 2′-deoxyuridine (FC = 2.26), thymidine 3′-monophosphate (FC = 2.44), and orotic acid (FC = 1.79) (Table S6), suggesting possible activation of both de novo and salvage pathways. Certain nucleotide metabolites (e.g., dAMP, 5-methyldeoxycytidine) increased intracellularly but decreased extracellularly (Table S6), which may reflect preferential retention of these metabolites within the cell. The concurrent increase in oxidative stress markers raises the possibility that salt-induced oxidative stress is associated with the observed changes in nucleotide metabolism, potentially to support nucleic acid repair; however, direct measurements of reactive oxygen species, glutathione status, and DNA or RNA repair activity would be required to confirm this interpretation. Collectively, these systemic physiological responses illuminate the metabolic trade-off observed in *E. lactis* KUST2812 (Figure 1E,F). The strain sacrifices cellular proliferation and diverts significant energy and resources toward maintaining osmotic balance, repairing stress-induced damage, and hyper-activating specific catabolic pathways, such as tyrosine catabolism, to ensure survival. This metabolic prioritization strategy aligns with the general principle of stress-induced resource reallocation observed across diverse bacterial taxa [40,41]. In contrast to the enhanced production of compatible solutes or exopolysaccharides reported in other halophiles under salt stress, the present findings suggest a distinctive mode of stress adaptation—the activation of a specific amino acid catabolic pathway to support energy and nitrogen demands. This contributes to our understanding of the diversity of microbial stress responses and highlights the metabolic versatility of food-derived Enterococcus strains under high-salt conditions.

### 3.9. Application in BBP Fermentation

Tyramine, a biogenic amine, poses a potential food safety concern due to its vasoactive effects in susceptible individuals [54]. Our multi-omics analysis suggested that while *E. lactis* KUST2812 efficiently degrades tyrosine, it may simultaneously convert a portion of tyrosine to tyramine. To evaluate this trade-off in a real food matrix, the strain was inoculated into a BBP fermentation system, and the changes in tyrosine and tyramine contents were monitored. The concentrations of tyrosine and tyramine in FBPJ are presented in Table 3. After 15 days of fermentation, both the naturally fermented group (NF) that served as the time-matched uninoculated control and the group inoculated with *E. lactis* KUST2812 (El) showed increased tyrosine levels compared to the initial control group (C). This increase may be attributed to the transfer of BBP samples to the laboratory environment, where altered conditions stimulated microbial metabolic activity, leading to enhanced proteolysis of the broad bean matrix and consequent tyrosine accumulation. Nevertheless, over the 15-day fermentation period, the net tyrosine accumulation in the El group (0.17 g/kg) was numerically lower than that in the NF group (0.36 g/kg), representing a 0.19 g/kg reduction in tyrosine buildup relative to the initial baseline.

The tyramine concentration in the El group was similar to that in the NF group at the endpoint (386.84 ± 11.72 vs. 386.67 ± 3.10 mg/kg). However, endpoint measurements alone cannot distinguish whether this reflects low tyramine production by the inoculated strain, degradation by the native microbial community, or other factors; further time-course studies are needed to clarify the underlying dynamics.

The reduced efficacy in BBP compared with liquid MRS medium is attributable to several factors: higher salinity (12–18% vs. 6% NaCl), the continuous dissolution of tyrosine from the broad bean substrate into the fermentation broth during the fermentation process (which accounts for the observed increase in tyrosine content in both the NF and El groups), and the complex microbial community that may compete with or constrain the inoculated strain. Nevertheless, the numerical reduction in net tyrosine accumulation observed in the El group suggests that *E. lactis* KUST2812 may retain activity under authentic BBP conditions. These findings may be viewed as preliminary observations for the strain’s activity in a laboratory-scale BBP system, rather than a demonstrated industrial application. The mechanistic experiments were designed to serve as a controlled model system for elucidating the regulatory mechanism, whereas the BBP validation at 12–18% NaCl suggests that the strain retains tyrosine catabolism activity under conditions closer to those encountered in practice. The BBP fermentation trial showed a numerical trend toward lower tyrosine accumulation in the El group without causing a notable increase in tyramine. Further evaluation under conditions that better reflect commercial fermentation (e.g., larger scale and complex native microbial communities) is needed to assess its industrial potential.

## 4. Conclusions

This study highlights the high halotolerance and unique metabolic capabilities of *E. lactis* KUST2812, suggesting its potential as a novel candidate for reducing tyrosine accumulation in high-salinity fermented foods. Our data suggest a distinct metabolic trade-off wherein salt stress suppresses bacterial proliferation but drastically enhances tyrosine catabolism. Multi-omics data suggest that this phenomenon is associated with the synergistic upregulation of the *tdc*–*tyrP* transport-conversion module and *tyrS*-mediated protein synthesis, coupled with extensive global osmotic stress responses. A preliminary BBP fermentation trial showed a numerical trend toward lower tyrosine accumulation in the El group without a marked increase in tyramine, suggesting a preliminary safety profile that warrants further investigation. The observation that the El group did not exhibit increased tyramine accumulation, together with the numerically reduced tyrosine levels, suggests that complex microbial interactions in the BBP ecosystem may influence tyramine turnover. Elucidating the specific mechanisms and responsible microbial taxa underlying this tyramine turnover represents a promising direction for future research.

Despite these promising findings, direct biochemical validation of the proposed molecular mechanisms—including in vitro assays of tyrosine decarboxylase activity under varying salinities, gene knockout strains (*Δtdc*, *ΔtyrP*), isotope tracing, and direct pathway-product measurements—remains necessary. Future work employing a factorial design to separate salt stress from secondary effects of substrate depletion and growth phase will help distinguish primary regulatory responses from downstream consequences. Co-cultivation with amine-degrading microorganisms should also be explored to optimize strain safety and performance.

## Figures and Tables

**Table 1:** Salt-tolerance-related genes identified in the genome of *Enterococcus lactis* KUST2812.

Category	Gene	Function
Ion transport	*nhaC*	Na^+^/H^+^ antiporter NhaC
	*nhaK*	Sodium:proton antiporter
	*napA*	Monovalent cation:proton antiporter-2 (CPA2) family protein
	*mdtG*	Multidrug efflux MFS transporter
	*zupT*	ZIP family metal transporter
	*czcD*	Cation diffusion facilitator family transporter
	*kup*	KUP/HAK/KT family potassium transporter
	*trkA*	Cation-translocating P-type ATPase
	*trkH*	TrkH family potassium uptake protein
	*clcA*	ClC family H(^+^)/Cl(^−^) exchange transporter
	*ATP2C*	Cation-translocating P-type ATPase
	*ctpE*	Cation-translocating P-type ATPase
	*ntpI*	V-type ATP synthase subunit K
	*zntA*	Heavy metal translocating P-type ATPase
	*yesM*	Histidine kinase
Membrane structure and modification	*plsY*	Glycerol-3-phosphate 1-O-acyltransferase PlsY
	*dapE*	ArgE/DapE family deacylase
	*clsA_B*	Cardiolipin synthase
	*ldh*	3-hydroxyacyl-CoA dehydrogenase NAD-binding domain-containing protein
Osmolyte synthesis and transport	*bceB*	ABC transporter permease
	*opuBD*	ABC transporter permease
	*glnQ*	Amino acid ABC transporter ATP-binding protein
	*glnH*	Amino acid ABC transporter substrate-binding protein/permease
	*gldA*	Glycerol dehydrogenase
	*gadC*	Glutamate:gamma-aminobutyrate antiporter
	*gadB*	Glutamate decarboxylase
	*proA*	Glutamate-5-semialdehyde dehydrogenase
	*proB*	Glutamate 5-kinase
	*oppB*	ABC transporter permease
	*fruA*	Fructose-specific PTS transporter subunit EIIC
	*proV*	Glycine betaine/L-proline ABC transporter ATP-binding protein
	*proW*	ABC transporter permease/substrate binding protein
	*opuA*	Betaine/proline/choline family ABC transporter ATP-binding protein
Signal transduction	*dacA*	Diadenylate cyclase CdaA
	*arlR*	Response regulator transcription factor
	*arlS*	HAMP domain-containing histidine kinase
Stress response and protein homeostasis	*dps*	DNA starvation/stationary phase protection protein
	*dnaK*	Molecular chaperone DnaK
	*dnaJ*	Molecular chaperone DnaJ
	*hslO*	Hsp33 family molecular chaperone HslO
	*btuE*	Glutathione peroxidase
	*tktA*	Universal stress protein
	*uspA*	Universal stress protein
	*nfrA1*	NADPH-dependent oxidoreductase

**Table 2:** Expression levels of key genes in *E. lactis* KUST2812 under 0 and 6% NaCl treatment.

Category	Gene	Expression Level		FC	Function
		C	S		
Tyrosine metabolism	*tdc*	0.75 ± 0.06	1.39 ± 0.17 *	1.85	Tyrosine decarboxylase, catalyzes conversion of tyrosine to tyramine
	*tdc*	14.26 ± 0.63	92.62 ± 1.08 ***	6.50	
	*tyrP*	7.49 ± 0.46	37.38 ± 1.31 ***	4.99	Tyrosine-specific transporter, enhances tyrosine uptake and tyramine efflux
	*tyrS*	194.99 ± 1.56	961.57 ± 43.85 ***	4.93	Tyrosyl-tRNA synthetase, channels tyrosine into protein synthesis
Salt tolerance	*nhaC*	2.68 ± 0.32	8.75 ± 0.53 ***	3.26	Na^+^/H^+^ antiporter NhaC
	*nhaC*	8.13 ± 0.73	72.91 ± 2.20 ***	8.97	
	*proV*	3.95 ± 0.46	136.73 ± 4.49 ***	34.62	Glycine betaine/L-proline ABC transporter ATP-binding protein
	*proW*	3.29 ± 0.35	370.85 ± 14.57 ***	112.72	
	*trkA*	40.99 ± 0.35	71.35 ± 3.44 **	1.74	Cation-translocating P-type ATPase
	*trkH*	4.30 ± 0.18	10.11 ± 0.42 **	2.35	TrkH family potassium uptake protein
	*clcA*	7.73 ± 0.89	25.55 ± 1.43 **	3.31	Voltage-gated chloride channel family protein
	*zntA*	1.12 ± 0.24	10.93 ± 0.05 ***	9.76	Heavy metal translocating P-type ATPase
	*gadC*	7.49 ± 0.46	37.38 ± 1.31 ***	4.99	Glutamate: gamma-aminobutyrate antiporter
	*oppD*	12.81 ± 0.42	27.73 ± 0.85 **	2.16	ABC transporter ATP-binding protein
	*oppA*	85.57 ± 5.76	145.43 ± 1.86 *	1.70	Peptide ABC transporter substrate-binding protein
	*oppB*	23.61 ± 2.89	49.87 ± 2.25 **	2.11	ABC transporter permease
	*fruA*	6.32 ± 0.80	100.45 ± 0.57 ***	15.89	Fructose-specific PTS transporter subunit EIIC
Cell growth	*ftsE*	71.34 ± 7.64	27.29 ± 0.90 **	0.38	Cell division ATP-binding protein FtsE
	*ftsX*	86.39 ± 3.59	95.57 ± 2.79 *	1.11	Permease-like cell division protein FtsX
Others	*treC*	1.12 ± 0.20	2.63 ± 0.26 *	2.35	α, α-Phosphotrehalase

Note: C, control group (0% NaCl); S, salt-stressed group (6% NaCl); FC, fold change (S vs. C). Values are presented as mean ± standard deviation. Significance levels: “*” corresponds to *p* < 0.05, “**” corresponds to *p* < 0.01, “***” corresponds to *p* < 0.001.

**Table 3:** Tyrosine and tyramine contents in fermented broad bean paste after 15 days of fermentation.

Sample	Tyrosine Content (g/kg)	Tyramine Content (mg/kg)
C	1.71 ± 0.20 ^b^	389.73 ± 5.12 ^a^
NF	2.07 ± 0.11 ^a^	386.67 ± 3.10 ^a^
El	1.88 ± 0.04 ^ab^	386.84 ± 11.72 ^a^

Note: C, initial control group; NF, naturally fermented group (without inoculation); El, group inoculated with *E. lactis* KUST2812. Data are presented as mean ± standard deviation. Within each column, different superscript letters indicate significant differences among groups *(p* < 0.05, one-way ANOVA followed by Holm-Šídák post-hoc test); identical letters indicate no significant difference (*p* > 0.05).

## Data Availability

The raw genome and transcriptome sequencing data and the genome assembly have been deposited in the NCBI Sequence Read Archive (SRA) under BioProject accession no. PRJNA1521597 (https://www.ncbi.nlm.nih.gov/bioproject/PRJNA1521597, accessed on 31 August 2026). The metabolomics raw data have been deposited in the Genome Sequence Archive (GSA) at the National Genomics Data Center (NGDC), China National Center for Bioinformation, under BioProject accession no. PRJCA073563 (https://ngdc.cncb.ac.cn/gsa/, accessed on 31 August 2026). All datasets will be released upon publication.

## Supplementary Materials

The following supporting information can be downloaded at: https://www.mdpi.com/article/10.3390/foods15183190/s1, Table S1: Composition of the enrichment medium; Table S2: Primers used for RT-qPCR; Table S3: Antibiotic susceptibility profile; Table S4: Tyrosine catabolism kinetics under different NaCl concentrations; Table S5: RNA-seq data quality summary; Table S6: Complete list of differentially expressed metabolites; Figure S1: Circular genome map of *E. lactis* KUST2812; Figure S2: KEGG functional annotation of the genome; Figure S3: Amplification curves (A) and melting peaks (B) of RT-qPCR. (A) Amplification plots showing the fluorescence signal versus cycle number for each gene. (B) Melting peak analysis displaying the negative first derivative of fluorescence (-dF/dT) as a function of temperature. A single sharp peak for each gene indicates specific amplification without primer dimers or non-specific products.

## Abbreviations

The following abbreviations are used in this manuscript:

BBP	Broad bean paste
UF	Uninoculated blank control
C	Control group
S	Salt-stressed group
E	Extracellular fraction
E6	Extracellular fraction under 6% NaCl stress
I	Intracellular fraction
I6	Intracellular fraction under 6% NaCl stress
DEGs	Differentially expressed genes
DEMs	Differentially expressed metabolites
El	*E. lactis*-inoculated group
NF	Naturally fermented group
EPS	Exopolysaccharides
IAA	Indole-3-acetic acid
FBPJ	Fermented broad bean paste juice
FC	Fold change
HPLC	High-performance liquid chromatography
