# Supplementary material for: Salt Stress Accelerates Tyrosine Depletion by Enterococcus lactis KUST2812: Multi-Omics Insights into Metabolic Remodeling
Source: Foods. 2026 Sep 9;15(18):3190. doi: 10.3390/foods15183190 (PMC13605990; doi:10.3390/foods15183190)
Supplement: Supplementary file 1 [file foods-15-03190-s001.zip › foods-4526095-supplementary.pdf]

Multi-omics reveals how salt activation mitigates tyrosine white spots formation in *Enterococcus lactis* from broad bean paste

Xiaoqi Gong[1,3,4], Qingyu Ma [2], Yujie Zhong[1,3,4], Zhijia Liu[1,3,4], Chuanqi Chu[1,3,4], Junjie Yi[1,3], Tao, Wang[1,3,4*]

1 Faculty of Food Science and Engineering, Kunming University of Science and Technology, Kunming 650500, China

2 School of Biological and Food Engineering, Honghe University, Mengzi 661199, China

3 Yunnan Key Laboratory of Plateau Food Intelligent Processing, Kunming 650500, China

4 International Green Food Processing Research and Development Center of Kunming City, Kunming 650500, China

* Corresponding author: Tao Wang (wtao_2017@163.com)

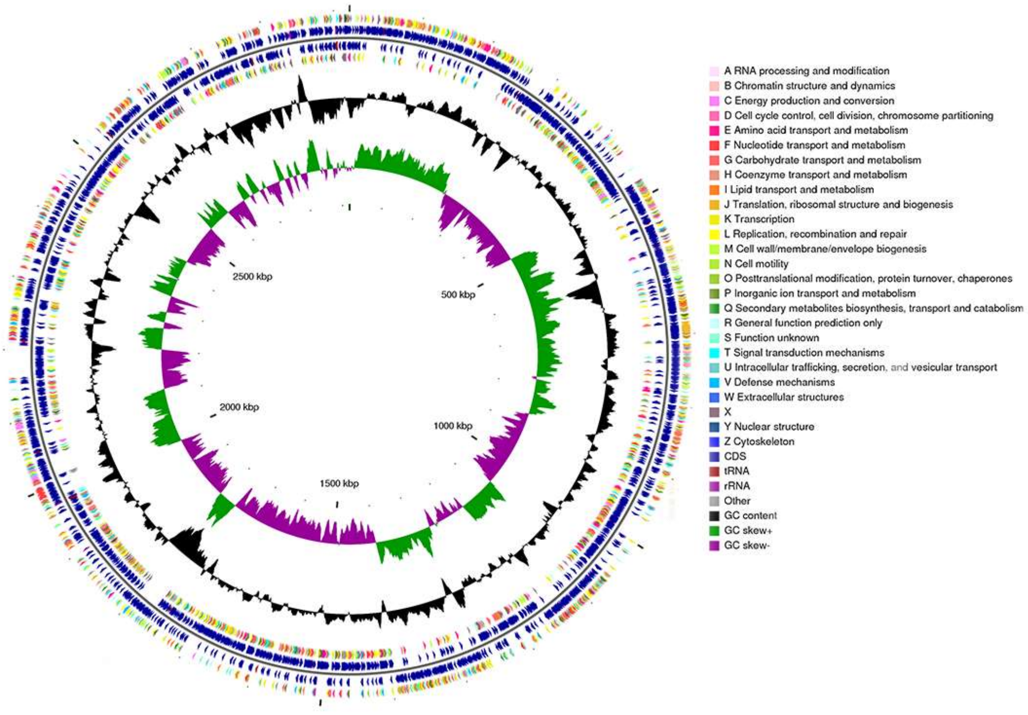

Figure S1 Circular genome map of *Enterococcus lactis* KUST2812 (from outer to inner circles: genome size, forward CDS, reverse CDS, rRNA and tRNA, GC content, and GC skew).

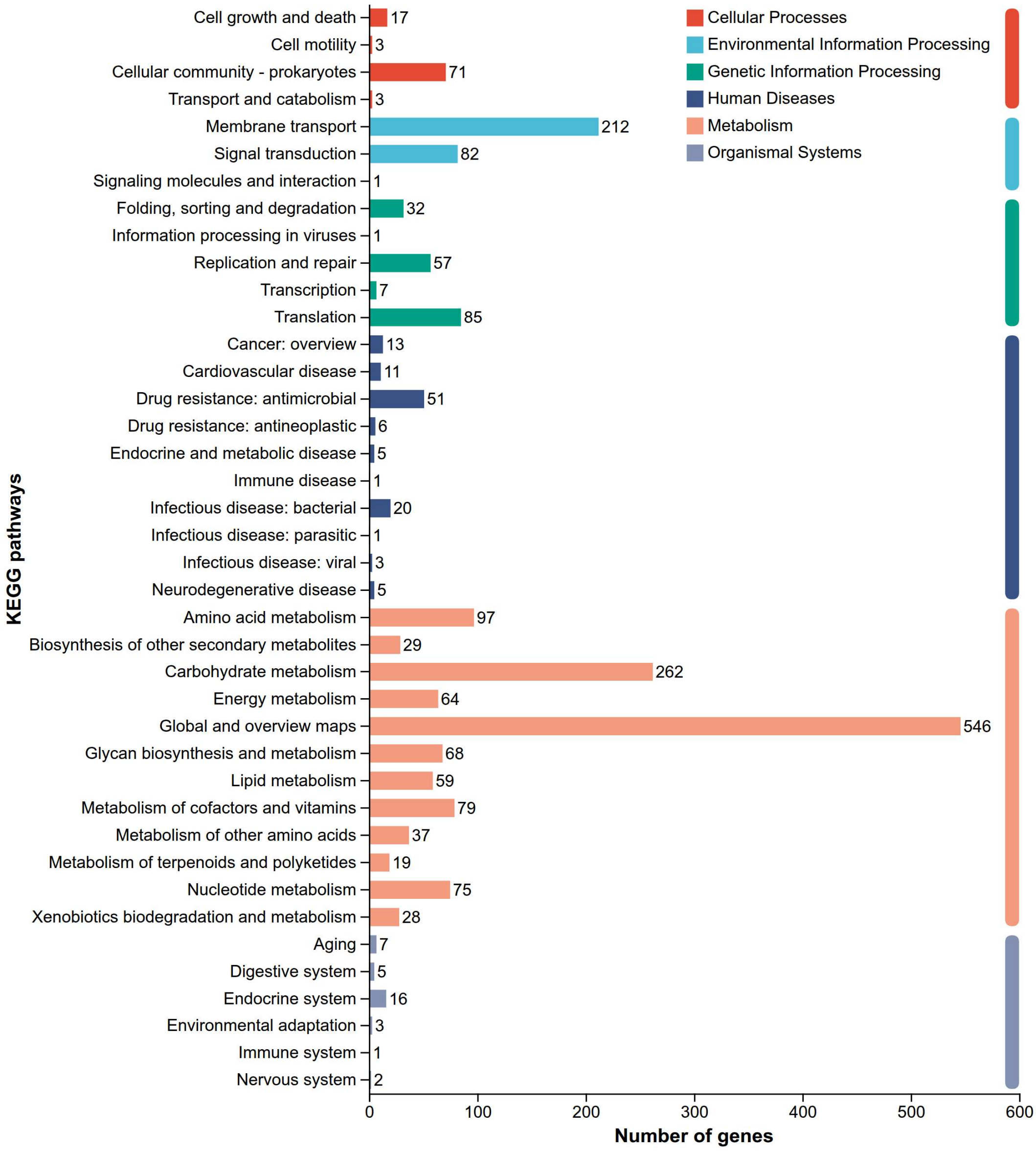

Figure S2 KEGG functional annotation of the *Enterococcus lactis* KUST2812 whole genome.

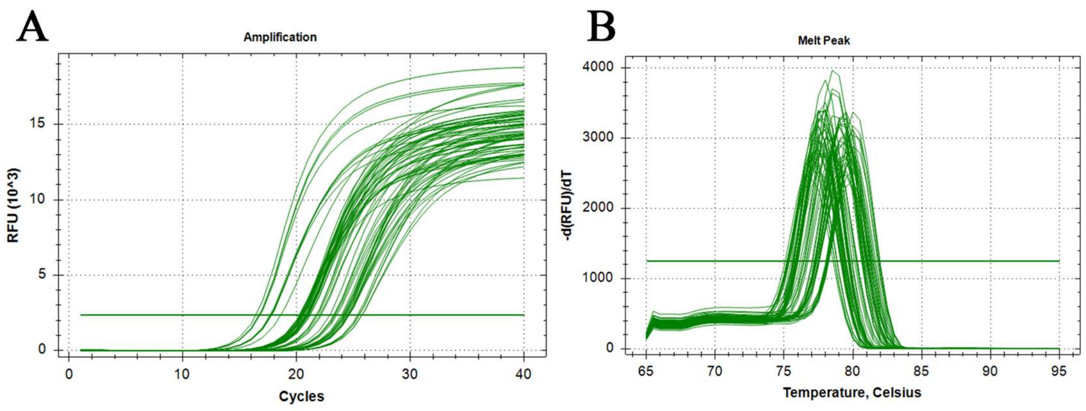

Figure S3 Amplification curves (A) and melting peaks (B) of RT-qPCR. (A) Amplification plots

showing the fluorescence signal versus cycle number for each gene. (B) Melting peak analysis displaying the negative first derivative of fluorescence (-dF/dT) as a function of temperature. A single sharp peak for each gene indicates specific amplification without primer dimers or non-specific products.

Table S1. Composition of the enrichment medium for halotolerant bacterial isolation.

**Component**	**Concentration (per liter)**
NaCl	100.0 g
Peptone	1.0 g
Beef extract	1.0 g
Yeast extract	1.0 g
Glucose	1.0 g
Tween-80	1.0 g
K_2HPO_4	2.0 g
Anhydrous sodium acetate	5.0 g
Sodium citrate	2.0 g
$MgSO_4 \cdot 7H_2O$	0.2 g
$MnSO_4 \cdot 5H_2O$	0.054 g

Preparation: All components were dissolved in distilled water, and sterilized by autoclaving at 115°C for 20 min.

Table S2. Primers used for RT-qPCR analysis.

**Gene**	**Primer sequence (5’→3’)**
map	F: CAGGTTGTTCCTGCGCTTTG<br>R: TAGGTGCTGGAAAAACGGGT
tdc	F: CAGACTATGGCAACAGCCCA<br>R: ATGGTGTCATTACGCTGGCA
tyrP	F: GGGGATTTGTCGTTGCATGG<br>R: GATGTTCCCGCCGACTTTTG
tyrS	F: AGAAACGTGAAGCACAACGC<br>R: TACCGAATCCTTGCGCGATT

proV	F: TACTGCCTGAAGTTGGCTCG<br>R: ACCAGCCGAAAACGGAGATT
proW	F: GGATCGTGTTTTCAGCAGCC<br>R: CGGTTTAGCAGTACCAGCCT
nhaC	F: TGTGCATGGAAAAAGGTGCC<br>R: GATTCGATGACCCCAGCCAA

Table S3. Antibiotic susceptibility of *E. lactis* KUST2812.

**Antibiotic**	**Potential resistance-associated genes**	**Identity (%)**	**Coverage (%)**	**Inhibition zone diameter (mm)**	**Susceptibility**
Penicillin	*PBP2x, PBP1a, PBP2b*	30.0-60.0	79.8-95.1	29.0 ± 3.7	S
Ampicillin				28.9 ± 2.6	S
Streptomycin	*AAC(6')-Ii*	98.9	99.5	14.6 ± 0.6	I
Tetracycline	*tetA(60), tetA(58), tet(42), tetA(46), rpsJ/poxtA*	22.7-65.7	17.9-97.6	43.9 ± 1.7	S
Doxycycline				47.2±0.6	S
Erythromycin	*msrC, efmA,efrA*	98.7	99.8	12.5 ± 2.9	R
Vancomycin	*vanR, vanS, vanH, vanZ, vanA, vanB*	25.6-51.1	25.4-97.8	26.0 ± 0.9	S
	D-Ala-D-Ala ligase	82.2	98.3		
Polymyxin B	*PmrF, arnA*	23.9-30.5	25.4-51.6	-	R
Cefazolin	*efmA, efrA*	100	99.8	17.6±1.4	I
Ceftazidime				-	R

Note: S, sensitive; I, intermediate; R, resistant; "–", no inhibition zone detected. Data are presented as mean ± standard deviation. Identity (%): > 70%, high homology (likely functional); 30–70%, moderate homology (potentially similar function); < 30%, low homology (weak functional relevance). Coverage %: > 90%, near-complete coverage (full function likely retained); 50–90%, partial coverage (function potentially incomplete); < 50%, insufficient coverage (unlikely to form a functional protein).

Table S4. Tyrosine concentrations (mg/L) during degradation kinetics under different NaCl concentrations.

Time	0%	1%	2%	3%	4%	5%	6%	7%	8%	9%	10%
0 h	626.02±6.14^{a}	627.14±10.01^{a}	625.99±5.31^{a}	642.74±9.10^{a}	644.02±6.92^{a}	619.75±3.00^{a}	606.75±3.42^{a}	612.97±2.86^{a}	622.27±6.74^{a}	602.11±5.29^{a}	618.38±3.44^{a}
6 h	409.83±22.20^{d}	450.18±31.37^{c}	187.13±17.59^{f}	75.21±2.92^{g}	105.51±11.79^{g}	161.36±2.51^{f}	341.68±11.19^{e}	537.12±46.90^{b}	595.80±5.07^{a}	575.64±3.97^{a}	606.53±5.23^{a}
12 h	214.66±28.65^{d}	180.22±15.27^{e}	ND	ND	ND	ND	ND	158.09±2.60^{f}	452.06±2.14^{c}	522.99±1.74^{b}	568.22±23.39^{a}
1 d	106.08±11.40^{c}	71.08±22.92^{e}	ND	ND	ND	ND	ND	ND	90.60±0.45^{d}	282.20±2.88^{b}	548.62±10.10^{a}
2 d	48.78±5.41^{c}	ND	ND	ND	ND	ND	ND	ND	ND	105.82±5.01^{b}	478.68±5.76^{a}
4 d	ND	ND	ND	ND	ND	ND	ND	ND	ND	ND	296.22±17.75^{a}
7 d	ND	ND	ND	ND	ND	ND	ND	ND	ND	ND	264.16±8.28^{a}
12 d	ND	ND	ND	ND	ND	ND	ND	ND	ND	ND	176.84±12.95^{a}

Note: Data are presented as mean ± SD. Within each row, different superscript letters indicate significant differences among NaCl concentrations at the same time point ($p <$ 0.05, one-way ANOVA followed by Holm–Šidák post-hoc test). ND, not detected.

Table S5. Quality assessment of sequencing data.

Sample Name	Clean Reads	Clean Bases (bp)	Clean Error Rate (%)	Clean Q20(%)	Clean Q30(%)
C1	29862680	4444597002	0.0112	99.55	97.31
C2	22437786	3346104613	0.0113	99.52	97.12
C3	22178402	3312002608	0.0112	99.55	97.36
S1	25748972	3841519741	0.0113	99.5	97.07
S2	23972236	3565620333	0.0113	99.51	97.22
S3	26764330	3974595699	0.0113	99.51	97.19

Note: C, samples from the control group (0% NaCl); S, samples from the salt-stressed group (6% NaCl).

Table S6. Abundance of key metabolites in intracellular and extracellular fractions of *Enterococcus lactis* KUST2812 under 0 and 6% NaCl treatment for 12 h.

Metabolite	Intracellular fraction (I6/I)						Extracellular fraction (E6/E)					
	FC	log_2FC	*p* value	VIP	Significant	Trend	FC	log_2FC	*p* value	VIP	Significant	Trend
L-Tyrosine	0.96	-0.05	2.10×10^{-1}	0.71	no	down	0.8	-0.32	1.11×10^{-5}	2.42	yes	down
Tyramine	1.05	0.07	1.30×10^{-3}	0.73	no	up	1.39	0.48	3.08×10^{-4}	2.18	yes	up
Proline	1.03	0.05	1.33×10^{-5}	1.04	yes	up	0.96	-0.06	1.16×10^{-4}	1.00	yes	down
Glutamate	1.24	0.31	3.75×10^{-7}	2.38	yes	up	0.93	-0.10	2.66×10^{-6}	1.25	yes	down
L-Citrulline	4.88	2.29	3.90×10^{-6}	4.21	yes	up	0.88	-0.19	1.59×10^{-5}	1.58	yes	down
Ornithine	1.28	0.36	7.52×10^{-8}	2.60	yes	up	0.84	-0.24	4.26×10^{-6}	1.84	yes	down
Aspartic Acid	1.09	0.13	9.53×10^{-8}	1.56	yes	up	0.9	-0.16	2.80×10^{-5}	1.50	yes	down
Threonine	1.04	0.06	3.58×10^{-4}	1.03	yes	up	0.94	-0.09	2.42×10^{-3}	1.04	yes	down
Isoleucine	1.04	0.06	3.37×10^{-5}	1.12	yes	up	0.99	-0.02	6.72×10^{-4}	0.60	no	down
Valine	1.18	0.24	1.26×10^{-8}	2.27	yes	up	0.97	-0.04	1.50×10^{-4}	0.87	no	down
Lysine	1.06	0.08	1.37×10^{-5}	1.27	yes	up	0.92	-0.13	3.70×10^{-5}	1.38	yes	down
Histidine	1.06	0.08	1.56×10^{-6}	1.22	yes	up	0.88	-0.18	3.89×10^{-5}	1.57	yes	down
4-Hydroxyproline	1.05	0.07	8.27×10^{-5}	1.16	yes	up	0.9	-0.15	6.17×10^{-6}	1.38	yes	down

Nalpha-Acetyl-L-Citrulline	2.58	1.37	2.08×10^{-9}	4.06	yes	up	1.16	0.21	1.09×10^{-2}	1.29	yes	up
Cysteinyl-Lysine	93.32	6.54	3.26×10^{-8}	4.79	yes	up	12.05	3.59	1.54×10^{-4}	3.24	yes	up
Udp-N-Acetyl-D-Galactosamine	0.4	-1.34	7.66×10^{-3}	4.25	yes	down	0.56	-0.83	8.23×10^{-4}	2.70	yes	down
8-Hydroxyguanosine	1.17	0.23	3.15×10^{-5}	1.80	yes	up	1.41	0.49	5.88×10^{-4}	2.18	yes	up
Alpha-Hydroxybutyric Acid	1.23	0.29	6.05×10^{-3}	1.75	yes	up	0.93	-0.10	5.70×10^{-6}	1.18	yes	down
Arabinose	1.12	0.17	5.82×10^{-4}	1.52	yes	up	0.95	-0.08	3.91×10^{-5}	1.04	yes	down
D-Arabinonic Acid	1.12	0.16	1.47×10^{-6}	1.61	yes	up	0.91	-0.13	1.93×10^{-4}	1.31	yes	down
D-(+)-Galactosamine	1.1	0.13	2.91×10^{-2}	1.21	yes	up	0.69	-0.54	1.64×10^{-6}	2.39	yes	down
L-Ribulose	1.07	0.10	6.87×10^{-2}	0.98	no	up	0.91	-0.14	1.96×10^{-4}	1.35	yes	down
Trehalose	0.7	-0.51	2.44×10^{-5}	2.61	yes	down	1.11	0.15	8.34×10^{-5}	1.44	yes	up
Adenosine 3',5'-Diphosphate	1.14	0.19	4.18×10^{-5}	1.92	yes	up	1.1	0.13	3.47×10^{-4}	1.39	yes	up
Thymidine 3'-Monophosphate	2.44	1.29	4.79×10^{-3}	3.55	yes	up	1.4	0.49	7.30×10^{-2}	1.54	no	up
2'-Deoxyuridine	2.26	0.53	4.69×10^{-3}	2.39	yes	up	1.07	0.10	1.68×10^{-4}	1.12	yes	up

Inosine	1.91	0.93	6.69×10^{-6}	3.60	yes	up	1.05	0.07	1.40×10^{-2}	0.92	no	up
Orotic Acid	1.79	0.84	3.68×10^{-8}	3.15	yes	up	1.21	0.28	3.87×10^{-6}	1.68	yes	up
Hypoxanthine	1.28	0.36	3.26×10^{-6}	2.45	yes	up	1	0.00	9.36×10^{-1}	0.03	no	-
2'-Deoxyinosine	1.26	0.34	1.34×10^{-4}	2.19	yes	up	1.01	0.01	3.80×10^{-1}	0.22	no	up
3-Deoxyuridine	1.26	0.34	8.95×10^{-6}	2.19	yes	up	1.09	0.12	2.12×10^{-6}	1.29	yes	up
2'-Deoxyadenosine 5'-Monophosphate	1.23	0.30	1.27×10^{-6}	2.46	yes	up	0.9	-0.15	8.76×10^{-3}	1.28	yes	down
Pseudouridine	1.13	0.17	9.13×10^{-4}	1.54	yes	up	1.11	0.15	1.76×10^{-5}	1.40	yes	up
Xanthosine	1.09	0.13	3.93×10^{-5}	1.48	yes	up	0.95	-0.08	7.69×10^{-5}	1.12	yes	down
5-Methyldeoxycytidine	1.07	0.10	1.70×10^{-1}	0.89	no	up	0.84	-0.26	2.31×10^{-4}	1.87	yes	down

Note: I6/I, ratio of intracellular metabolites in salt-stressed group (6% NaCl) to control group (0% NaCl); E6/E, ratio of extracellular metabolites in salt-stressed group (6% NaCl) to control group (0% NaCl). FC, fold change; VIP, variable importance in projection. Significance was determined based on $p < 0.05$ and VIP > 1.
